# Evaluation of an artificial intelligence noise reduction tool for conventional X-ray imaging – a visual grading study of pediatric chest examinations at different radiation dose levels using anthropomorphic phantoms

**DOI:** 10.1007/s00247-025-06251-0

**Published:** 2025-05-13

**Authors:** Maria Hultenmo, Johanna Pernbro, Jenny Ahlin, Martin Bonnier, Magnus Båth

**Affiliations:** 1 Department of Medical Physics and Biomedical Engineering, Sahlgrenska University Hospital, Gula stråket 2B, SE-413 45, Gothenburg, Sweden; 2 Department of Medical Radiation Sciences, Institute of Clinical Sciences, Sahlgrenska Academy, University of Gothenburg, Gothenburg, Sweden; 3 Department of Pediatric Radiology, Queen Silvia Children’s Hospital, Sahlgrenska University Hospital, Gothenburg, Sweden

**Keywords:** Artificial intelligence, Chest radiography, Dose reduction, Intelligent noise reduction, Pediatric, Radiation dose

## Abstract

**Background:**

Noise reduction tools developed with artificial intelligence (AI) may be implemented to improve image quality and reduce radiation dose, which is of special interest in the more radiosensitive pediatric population.

**Objective:**

The aim of the present study was to examine the effect of the AI-based intelligent noise reduction (INR) on image quality at different dose levels in pediatric chest radiography.

**Materials and methods:**

Anteroposterior and lateral images of two anthropomorphic phantoms were acquired with both standard noise reduction and INR at different dose levels. In total, 300 anteroposterior and 420 lateral images were included. Image quality was evaluated by three experienced pediatric radiologists. Gradings were analyzed with visual grading characteristics (VGC) resulting in area under the VGC curve (AUC_VGC_) values and associated confidence intervals (CI).

**Results:**

Image quality of different anatomical structures and overall clinical image quality were statistically significantly better in the anteroposterior INR images than in the corresponding standard noise reduced images at each dose level. Compared with reference anteroposterior images at a dose level of 100% with standard noise reduction, the image quality of the anteroposterior INR images was graded as significantly better at dose levels of ≥ 80%. Statistical significance was also achieved at lower dose levels for some structures. The assessments of the lateral images showed similar trends but with fewer significant results.

**Conclusion:**

The results of the present study indicate that the AI-based INR may potentially be used to improve image quality at a specific dose level or to reduce dose and maintain the image quality in pediatric chest radiography.

**Graphical abstract:**

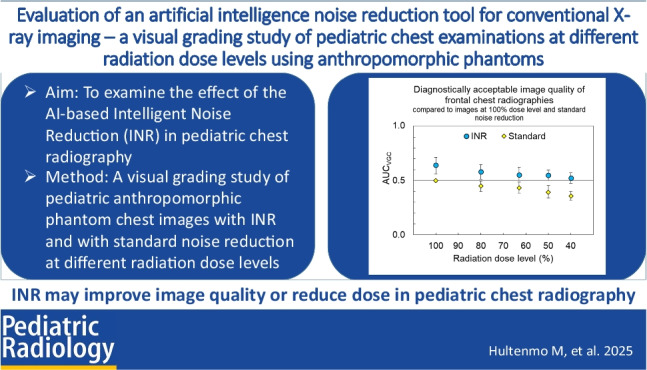

## Introduction

In recent years, advancements in artificial intelligence (AI) have played a major role in the development and evaluation of software for radiological applications. More than 200 AI-based tools have been European Conformity (CE)-marked or approved by the Food and Drug Administration (FDA) [1]. One of the subspecialties of radiology with the highest number of CE-marked AI software is chest radiology, where AI tools can be used for the detection of pathologies, e.g., lung nodules, pneumonia, and pneumothorax, in both chest radiography and chest computed tomography (CT) [2], and may lead to benefits such as earlier detection, increased diagnostic accuracy, and reduced reading time for radiologists [3].

Another important application of AI within chest radiology is image quality improvement. By training a deep neural network (DNN) to find the image characteristics of ground truth image data, e.g., high-dose data, it can achieve the knowledge of how to mimic corresponding high image quality when reconstructing low-dose data [4, 5]. In this way, noise-reducing deep learning reconstruction (DLR) algorithms that preserve a desired noise structure as well as spatial resolution can be developed. The use of these algorithms may allow the radiation dose to be reduced in specific cases without degrading the diagnostic image quality [6]. This is of special interest in the more radiosensitive pediatric population in which justification and optimization of radiographic examinations are of high importance [7].

Pediatric chest radiographs are frequently used for the detection of pathological conditions as well as the inspection of tube and line positions, e.g., in intensive care units [2]. However, the radiation dose associated with X-ray examinations is related to a stochastic risk of radiation-induced cancer, even at the generally low doses used in diagnostic radiology [7]. Children are more sensitive to ionizing radiation than adults, since their developing state of tissues increases the potential negative effects in cells. Furthermore, children have a longer remaining lifetime during which radiation-induced cancer can manifest after a latency period of years to several decades.

Although conventional chest examinations deliver low patient doses, several of the most radiosensitive organs, such as the lungs, breasts, and the red bone marrow [8], are exposed to ionizing radiation. Also, due to their frequent use, the contribution from chest radiographs to the pediatric collective radiation dose cannot be neglected [9]. Therefore, pediatric chest radiography is an important object of optimization and radiation dose assessments, e.g., with demands on the determination of diagnostic dose reference levels for different age- or weight intervals.

New possibilities for radiation dose reduction, such as AI noise reduction tools, are therefore of great interest in pediatric radiology [3, 6]. However, most currently available AI applications have not been developed and approved for specific pediatric use [2]. Detailed information about the image data used to train DNN, such as age, gender, and potential pathologies of the patients, is not always specified by vendors. Differences in both anatomy proportions and pathologies between children and adults potentially may affect the diagnostic benefits of AI tools. Also, the FDA approval process or CE marking of AI products considers the risk of injury, not the diagnostic value for the patients [3]. Therefore, AI software should be implemented carefully in radiology clinics to ensure that the desired image quality is achieved without the loss of diagnostic information or the introduction of artifacts in images. These concerns are especially important when introducing AI software in pediatric radiology.

According to a recent review by Ng, only 16 relevant studies have been published with the aim of evaluating AI post-processing applications for dose optimization in pediatric radiology [6]. Most of these involved studies of commercially available DLR tools in CT and were mostly based on patient images, although a few were based on phantom images or a combination of these. Only a single study by Krueger et al. on the use of AI in conventional pediatric radiography [10] fulfilled the criteria to be included in the review. Ng concluded that there is a need for further studies involving different modalities and examinations to investigate the dose optimization possibilities of AI tools within pediatric radiology [6].

The Intelligent Noise Reduction (INR) algorithm from Canon Medical Systems (Ōtawara, Japan) is a newly introduced AI-based tool [11–13]. The software is CE-marked and FDA-approved and available for Canon CXDI detectors within conventional radiography. About 3,000 clinical X-ray images were used in the DNN when developing INR with the aim of noise reduction with maintained anatomical details. However, the performance of the software in the pediatric population is unclear. Thus, the aim of the present study was to examine the effect of the AI-based INR on image quality at different dose levels in pediatric chest radiography. Specifically, a visual grading study was performed to assess the image quality of chest images obtained at different dose levels using anthropomorphic phantoms.

## Materials and methods

A visual grading study based on phantom images was performed to assess the effect of INR when applied in pediatric chest radiography at different exposure levels (corresponding to different patient dose levels). The phantom study simulated bedside chest radiography in anteroposterior position, which is generally used for younger pediatric patients that cannot co-operate to sitting or standing examinations. Bedside chest radiography is also applied for pediatric patients that are too sick to stand during examination or must be examined at another unit of the hospital, e.g., in intensive care.

### Phantoms

Two anthropomorphic phantoms, PBU-70 and PBU-60 (Kyoto Kagaku, Kyoto, Japan), were used to simulate chest examinations of pediatric patients of different sizes. According to manufacturer specifications, the phantoms represent patient sizes of 20 kg/110 cm and 50 kg/165 cm, respectively.

### Exposure settings

The exposure parameters were based on clinical settings of chest examinations of a conventional X-ray system Arcoma Precision i5 (Mediel, Mölndal, Sweden) equipped with Canon detectors at the Department of Pediatric Radiology at Queen Silvia Children’s Hospital, Sahlgrenska University Hospital, Gothenburg, Sweden. The pediatric radiological department performs approximately 30,000 X-ray based examinations each year, of which roughly 6,000 are conventional chest examinations. At the time of the study, INR was not installed in the clinic. Hence, the clinical protocols, including exposure and image processing settings, were implemented in an equivalent X-ray system at a vendor’s laboratory (Diagnosticum, Mediel, Mölndal, Sweden) where INR was installed, to reflect the image quality of the chest radiographies at the pediatric radiological department. The INR-compatible wireless detector CXDI-410C (Canon Medical Systems) was used in the study. The fixed tube load in the clinical settings was already set to the lowest achievable value (0.3 mAs) of the X-ray generator. To be able to reduce the dose level in a controlled way, a higher filtration as well as a longer source-to-detector distance (SDD) was applied. In this way, the tube load was increased initially and reduced to achieve different dose levels. However, the higher filtration increased the radiation quality (mean X-ray energy), resulting in increased penetration ability of the X-rays and a higher detector dose. This was compensated by choosing chest protocols of lower ages (0–1 and 2–3-year-old) which had lower tube voltages, i.e., lower radiation quality, as a base for the exposure settings for PBU-70 and PBU-60, respectively.

The standard 100% dose level was achieved by adjusting the initial tube load so that an exposure index of 150 was achieved. This results in sufficient diagnostic quality in chest images at the present pediatric radiological department. The exposure index of a digital X-ray radiographic image is an indicator of the detector dose in the anatomically relevant region and is proportional to the exposure of the detector when using a fixed radiation quality [14]. The anteroposterior and lateral images were exposed at dose reduction steps according to the basic Renard series R10 [15]: 100%, 80%, 63%, 50%, and 40% of the original dose. The lateral images were additionally exposed at dose levels of 32% and 25% of the original dose. From clinical experience, lower radiation doses may be used with acceptable image quality for lateral images. The exposure parameters are shown in Table 1.

**Table 1:** Exposure settings of the chest radiography for the two anthropomorphic phantoms (Kyoto Kagaku)

Phantom	PBU-70		PBU-60	
Tube voltage (kV)	81		102	
Filtration	1 mmAl + 0.2 mmCu		2 mmAl + 0.3 mmCu	
SDD (cm)	141		148	
Focus	Small		Small	
Tube load (mAs)^a^	Anteroposterior	Lateral	Anteroposterior	Lateral
100% [150]	0.8	2.0	0.8	2.2
80% [120]	0.6	1.6	0.6	1.8
63% [95]	0.5	1.2	0.5	1.4
50% [75]	0.4	1.0	0.4	1.1
40% [60]	0.3	0.8	0.3	0.9
32% [48]	-	0.6	-	0.7
25% [38]	-	0.5	-	0.5

^a^At a specific dose level [exposure index] and projection

### Image acquisition

For each exposure setting and projection, five images with slightly different tube angles (− 5°, − 3°, 0°, + 3°, and + 5°) were acquired to obtain different overlays and anatomical noise of the two phantoms’ organs and tissues, thereby simulating different patients. According to Hoeschen et al., the noise in chest radiographic images consists of both anatomical and technical noise [16]. The anatomical noise is different from the anatomical background, which is a relevant part of the clinical image and not described as noise. The anatomical noise is due to the many small anatomical structures that are overlayed along the X-ray path in the lung area and is described to increase the overall image noise. Even a minor change in radiation geometry generates a completely different anatomical noise pattern in a chest radiograph due to the differences in the overlay of small structures contributing to the final image. The technical noise includes detector noise and quantum noise, and the latter varies between each exposure due to the stochastic nature of X-rays. Hence, the acquisition of each image was also repeated three times to obtain variations in statistical noise, thereby affecting the image quality of the two phantoms’ organs and tissues. In total, 300 anteroposterior images and 420 lateral images were acquired and included in the study. The difference in number of images between projections was due to different number of dose levels, as described earlier.

### Postprocessing image noise reduction

**Fig. 1:**
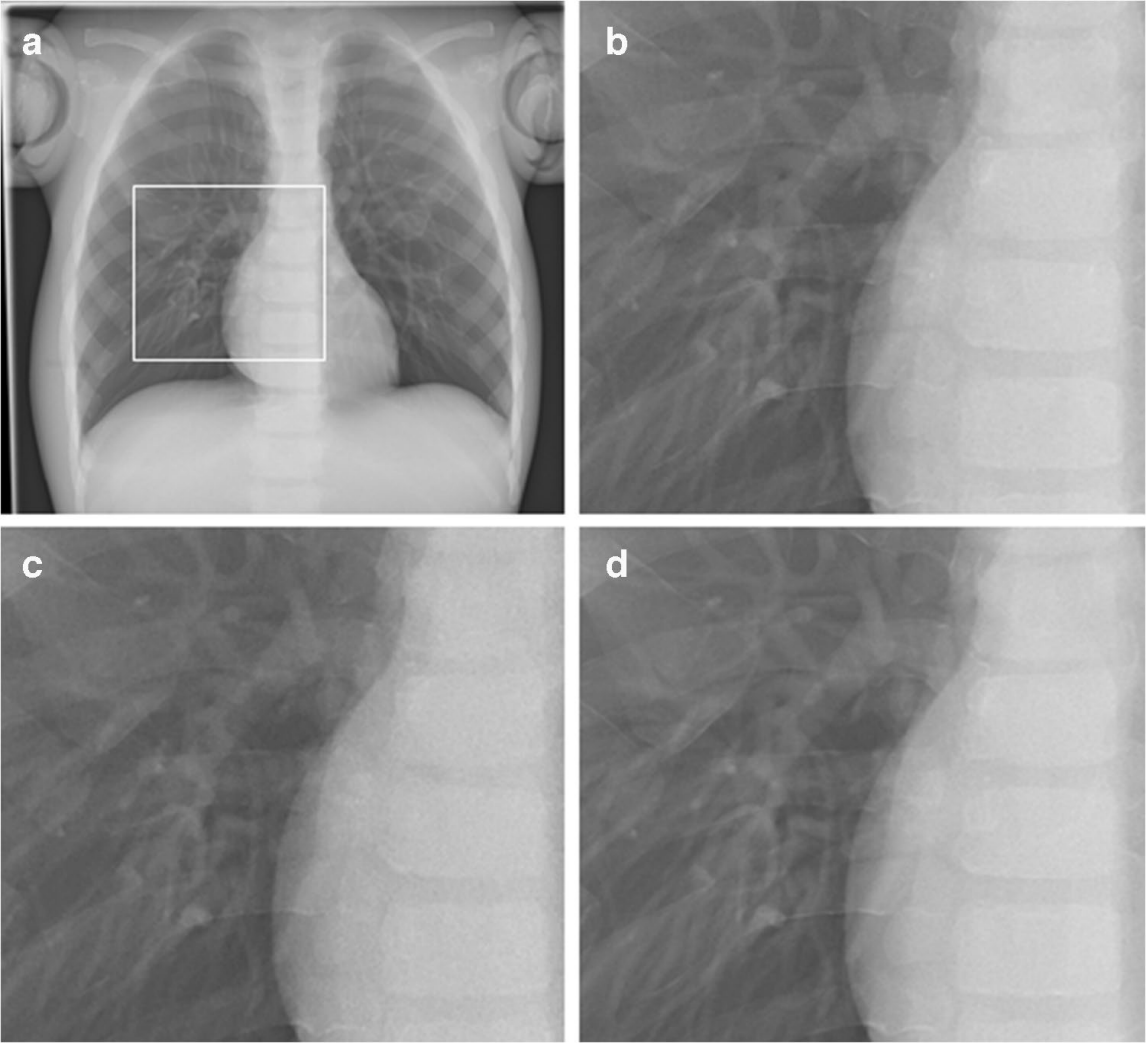
Anteroposterior chest radiographs of the PBU-70 phantom (Kyoto Kagaku). **a** Dose level 100% with standard noise reduction. The image shows the magnified region in images (**b**-**d**) (*box*). **b**-**d** Magnified images at dose level 100% with standard noise reduction (**b**), dose level 40% with standard noise reduction (**c**), and dose level 40% with intelligent noise reduction (**d**)

**Fig. 2:**
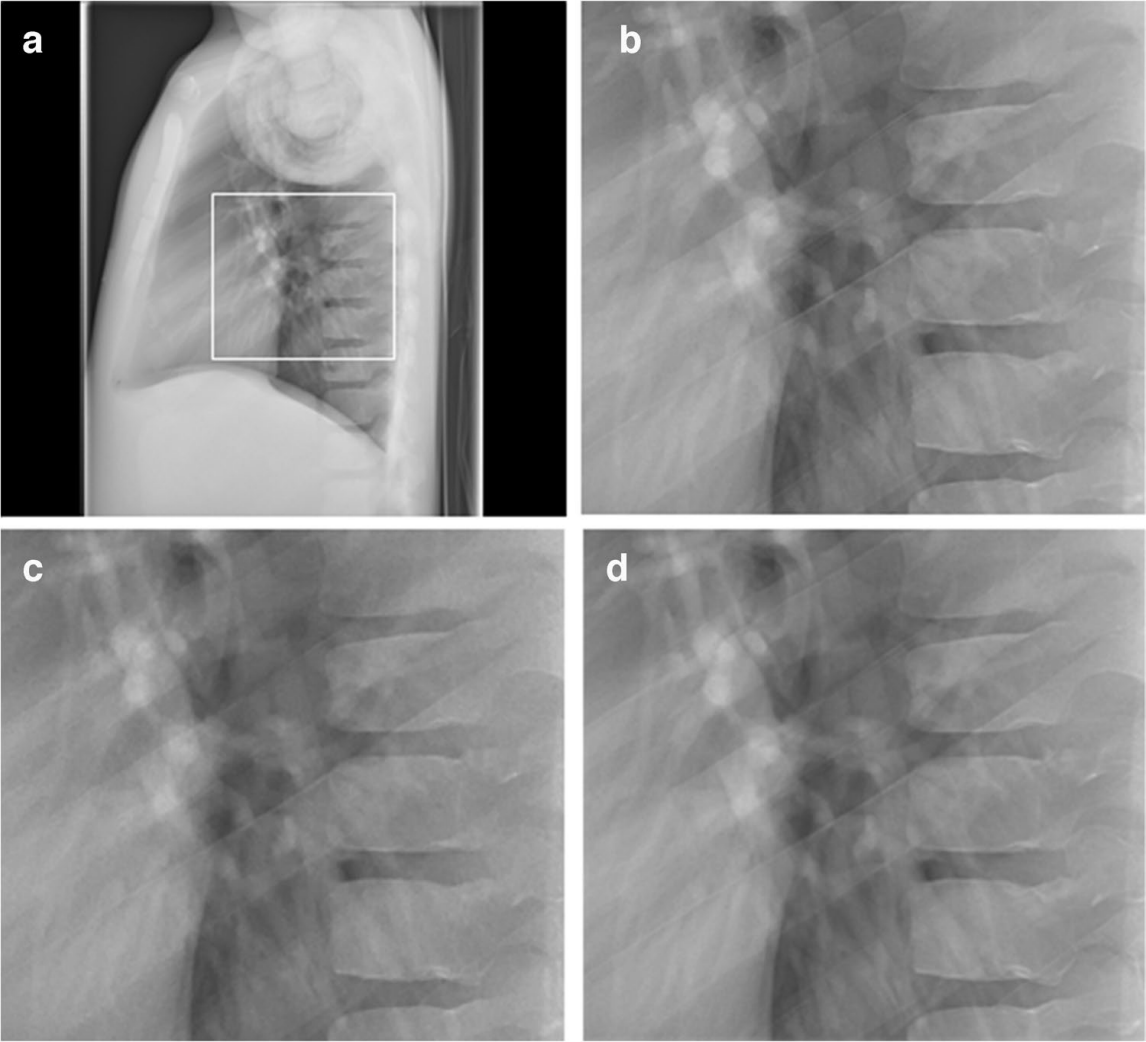
Lateral chest radiographs of the PBU-70 phantom (Kyoto Kagaku). **a** Dose level 100% with standard noise reduction. The image shows the magnified region in images **(b**-**d**) (*box*). **b**-**d** Magnified images at dose level 100% with standard noise reduction (**b**), dose level 25% with standard noise reduction (**c**), and dose level 25% with intelligent noise reduction (**d**)

Anteroposterior and lateral images of both phantoms were acquired with the routinely applied digital postprocessing (standard noise reduction) as well as with INR; see examples in Figs. 1 and 2. INR can be applied at a level of 1–10, ranging from the lowest (1) to the highest (10) influence of INR on noise reduction. In this study, a medium level of 5 was used for all images.

### Image grading

The image quality of the chest radiographs was evaluated in a visual grading study by three pediatric radiologists working at the Department of Pediatric Radiology at Queen Silvia Children’s hospital, Sahlgrenska University Hospital, Gothenburg, Sweden. Each radiologist had 8 years of experience. The software ViewDEX version 2.57 (Sahlgrenska University Hospital and University of Gothenburg, Gothenburg, Sweden) was used to show the phantom images in random order and fully blinded [17, 18]. The anteroposterior and lateral images were presented in two separate ViewDEX studies. The radiologists graded the quality of each image independently according to seven criteria for the anteroposterior images and five criteria for the lateral images. The criteria were based on the European guidelines on quality criteria for diagnostic radiographic images in pediatrics [19], with two additional general criteria regarding diagnostically acceptable image quality and clinically acceptable artifacts in the images (Table 2). The observers used a five-step ordinal scale (Table 3) to grade each criterion. Before each viewing session, the observers evaluated a small test set, including high, mid, and low radiation dose images with both INR and standard noise reduction, to calibrate their rating levels.

**Table 2:** Image quality criteria used for the anteroposterior and lateral chest radiographs in the visual grading study

Label	Criterion
*Anteroposterior*	
AP1	Clear reproduction of vascular pattern
AP2	Clear reproduction of the trachea and the proximal bronchi
AP3	Visually sharp reproduction of the diaphragm and costo-phrenic angles
AP4	Clear reproduction of the spine and paraspinal structures
AP5	Clear reproduction of the retrocardiac lung and the mediastinum
AP6	Clinically acceptable image quality for a standard lung exam
AP7	Clinically acceptable artifacts
*Lateral*	
L1	Visually sharp reproduction of the diaphragm and costo-phrenic angles
L2	Clear reproduction of the spine and paraspinal structures
L3	Clear reproduction of the retrocardiac lung and the mediastinum
L4	Clinically acceptable image quality for a standard lung exam
L5	Clinically acceptable artifacts

**Table 3:** Rating scale used by the radiologists in the visual grading study

Grade	Level of confidence
A	Confident that the criterion is fulfilled
B	Somewhat confident that the criterion is fulfilled
C	Indecisive whether the criterion is fulfilled or not
D	Somewhat confident that the criterion is not fulfilled
E	Confident that the criterion is not fulfilled

### Statistical analysis

The image gradings were evaluated with visual grading characteristics (VGC) analysis [20, 21]. VGC analysis is a statistical method used to analyze data from studies in which observers have rated the image quality on an ordinal scale, e.g., by stating how well certain criteria are fulfilled, like in the present study. The analysis generates a VGC curve describing the relationship between the rating distributions of a reference image set and a test image set. The figure of merit used in VGC analysis is the area under the VGC curve (AUC_VGC_), which spans from 0 to 1. The AUC_VGC_ is larger than 0.5 if the test image set is rated higher than the reference image set and the higher the AUC_VGC_ the larger the difference in image quality between the two image sets. In accordance, the AUC_VGC_ is smaller than 0.5 if the reference image set is rated higher than the test image set. The VGC analysis was performed with the software VGC Analyzer 1.0 version 3 (Sahlgrenska University Hospital and University of Gothenburg, Gothenburg, Sweden) [22–24]. VGC Analyzer is a software especially developed to perform the VGC analysis and apply appropriate statistical tests. VGC Analyzer calculates the AUC_VGC_ and its uncertainty, expressed as the 95% confidence interval (CI). A 95% CI that does not include 0.5 is considered to indicate a statistically significant difference in image quality between the two image sets. The uncertainty of the AUC_VGC_ was determined for the group of observers (fixed-reader analysis), and not generalized to the population of observers.

Two VGC analyses were performed. In the first analysis, the images with standard noise reduction were used as the reference image set and compared to the corresponding INR images at each examined dose level. In the second analysis, the images with standard noise reduction at 100% dose level, aiming at a clinical exposure index, were used as the reference image set and compared to all other image sets, i.e., differing in noise reduction method and dose level.

## Results

The VGC analysis showed that the experienced pediatric radiologists graded the image quality of the anteroposterior INR images as statistically significantly better than that of the standard noise reduced images at each examined dose level (40–100% of the reference dose). This was the case for all criteria (AP1-AP5) regarding important individual anatomical structures and the criterion of clinically acceptable image quality for a standard lung exam (AP6); see Table 4.

**Table 4:** Results of the visual grading characteristic (VGC) analysis described by the area under the VGC curve (AUC_VGC_) values from the study comparing anteroposterior chest test images with intelligent noise reduction (INR) to corresponding reference images with standard noise reduction at different dose levels and for different image quality criteria

Criterion	AUC_VGC_ (CI lower, CI upper) at specific radiation dose level				
	100%	80%	63%	50%	40%
AP1	**0.61** (0.57, 0.66)	**0.73** (0.68, 0.79)	**0.78** (0.72, 0.83)	**0.78** (0.72, 0.83)	**0.82** (0.76, 0.88)
AP2	**0.56** (0.52, 0.59)	**0.59** (0.56, 0.63)	**0.59** (0.54, 0.63)	**0.58** (0.55, 0.61)	**0.56** (0.52, 0.60)
AP3	**0.63** (0.59, 0.68)	**0.61** (0.56, 0.65)	**0.62** (0.57, 0.67)	**0.65** (0.60, 0.68)	**0.62** (0.57, 0.66)
AP4	**0.58** (0.53, 0.63)	**0.62** (0.57, 0.67)	**0.59** (0.54, 0.65)	**0.58** (0.55, 0.62)	**0.64** (0.58, 0.69)
AP5	**0.64** (0.56, 0.71)	**0.65** (0.58, 0.72)	**0.65** (0.60, 0.70)	**0.67** (0.61, 0.73)	**0.66** (0.60, 0.73)
AP6	**0.64** (0.56, 0.71)	**0.61** (0.57, 0.65)	**0.62** (0.58, 0.66)	**0.65** (0.60, 0.70)	**0.67** (0.63, 0.70)
AP7	0.51 (0.48, 0.54)	**0.54** (0.52, 0.57)	**0.55** (0.51, 0.58)	**0.57** (0.54, 0.60)	**0.59** (0.55, 0.62)

INR images were rated higher than images with standard noise reduction if AUC_VGC_ > 0.5, and lower if AUC_VGC_ < 0.5. Bold indicates statistical significance, i.e., when the confidence interval (CI) does not include 0.50. For explanation of each criterion, see Table 2

The corresponding VGC analysis for the lateral images (criteria L1-L4) favored INR for each dose level; see Table 5. However, statistically significant improvements in image quality were only obtained for anatomical structures at dose levels of ≤ 80% for criteria L2 and L3 and ≤ 63% for criterion L1. Statistically significant improvement of diagnostically acceptable image quality (criterion L4) was observed at dose levels of ≤ 50% and at 80%.

**Table 5:** Results of the visual grading characteristic (VGC) analysis described by the area under the VGC curve (AUC_VGC_) values from the study comparing lateral chest test images with intelligent noise reduction (INR) to corresponding reference images with standard noise reduction at different dose levels and for different image quality criteria

Criterion	AUC_VGC_ (CI lower, CI upper) at specific radiation dose level						
	100%	80%	63%	50%	40%	32%	25%
L1	0.52 (0.47, 0.58)	0.52 (0.47, 0.58)	**0.56** (0.51, 0.62)	**0.62** (0.57, 0.67)	**0.61** (0.55, 0.67)	**0.64** (0.58, 0.70)	**0.63** (0.57, 0.69)
L2	0.55 (0.50, 0.60)	**0.64** (0.58, 0.69)	**0.66** (0.59, 0.73)	**0.70** (0.64, 0.75)	**0.68** (0.62, 0.75)	**0.70** (0.64, 0.75)	**0.76** (0.70, 0.82)
L3	0.51 (0.44, 0.57)	**0.63** (0.57, 0.68)	**0.58** (0.52, 0.65)	**0.62** (0.57, 0.67)	**0.60** (0.54, 0.66)	**0.61** (0.57, 0.65)	**0.68** (0.63, 0.74)
L4	0.51 (0.47, 0.54)	**0.56** (0.53, 0.60)	0.52 (0.49, 0.55)	**0.55** (0.52, 0.58)	**0.56** (0.53, 0.59)	**0.57** (0.54, 0.60)	**0.60** (0.56, 0.63)
L5	0.53 (0.50, 0.56)	0.53 (0.50, 0.56)	0.53 (0.50, 0.56)	**0.55** (0.51, 0.59)	0.52 (0.49, 0.55)	**0.56** (0.53, 0.59)	**0.57** (0.54, 0.61)

INR images were rated higher than images with standard noise reduction if AUC_VGC_ > 0.5, and lower if AUC_VGC_ < 0.5. Bold indicates statistical significance, i.e., when the confidence interval (CI) does not include 0.50. For explanation of each criterion, see Table 2

**Fig. 3:**
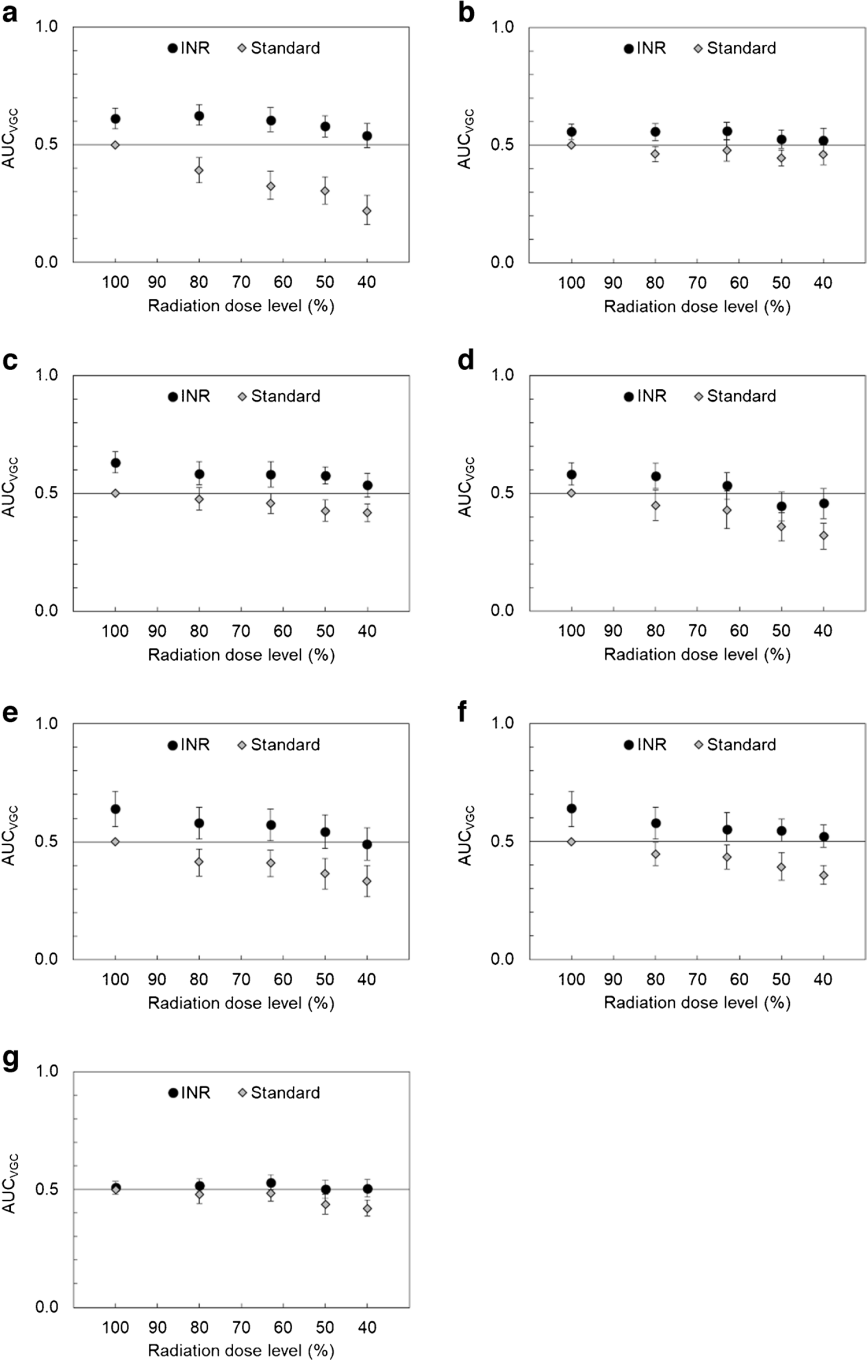
Results of the visual grading characteristic (VGC) analysis for the study comparing anteroposterior chest test images, with intelligent noise reduction (INR) as well as with standard noise reduction, at different dose levels to corresponding reference images (dose level 100% with standard noise reduction). Test images were rated higher than reference images if the area under the VGC curve (AUC_VGC_) > 0.5, and lower if AUC_VGC_ < 0.5. The results were statistically significant if the confidence interval (CI), indicated with *error bars*, did not include 0.5. **a** Clear reproduction of vascular pattern (criterion AP1). **b** Clear reproduction of the trachea and the proximal bronchi (criterion AP2). **c** Visually sharp reproduction of the diaphragm and costo-phrenic angles (criterion AP3). **d** Clear reproduction of the spine and paraspinal structures (criterion AP4). **e** Clear reproduction of the retrocardiac lung and the mediastinum (criterion AP5). **f** Clinically acceptable image quality for a standard lung exam (criterion AP6). **g** Clinically acceptable artifacts (criterion AP7)

In the second VGC analysis, the image quality of the anteroposterior chest images with INR was graded as significantly higher for all criteria AP1-AP6 at dose levels of ≥ 80% compared to the reference anteroposterior images with standard noise reduction and 100% dose level. Statistical significance was also achieved for the INR images at lower dose levels: ≥ 63% for criteria AP2 and AP5 and ≥ 50% for criteria AP1 and AP3; see Fig. 3. The gradings of the INR images at 40% of the reference dose were not significantly different from those of the reference images for any of the criteria AP1-AP7.

**Fig. 4:**
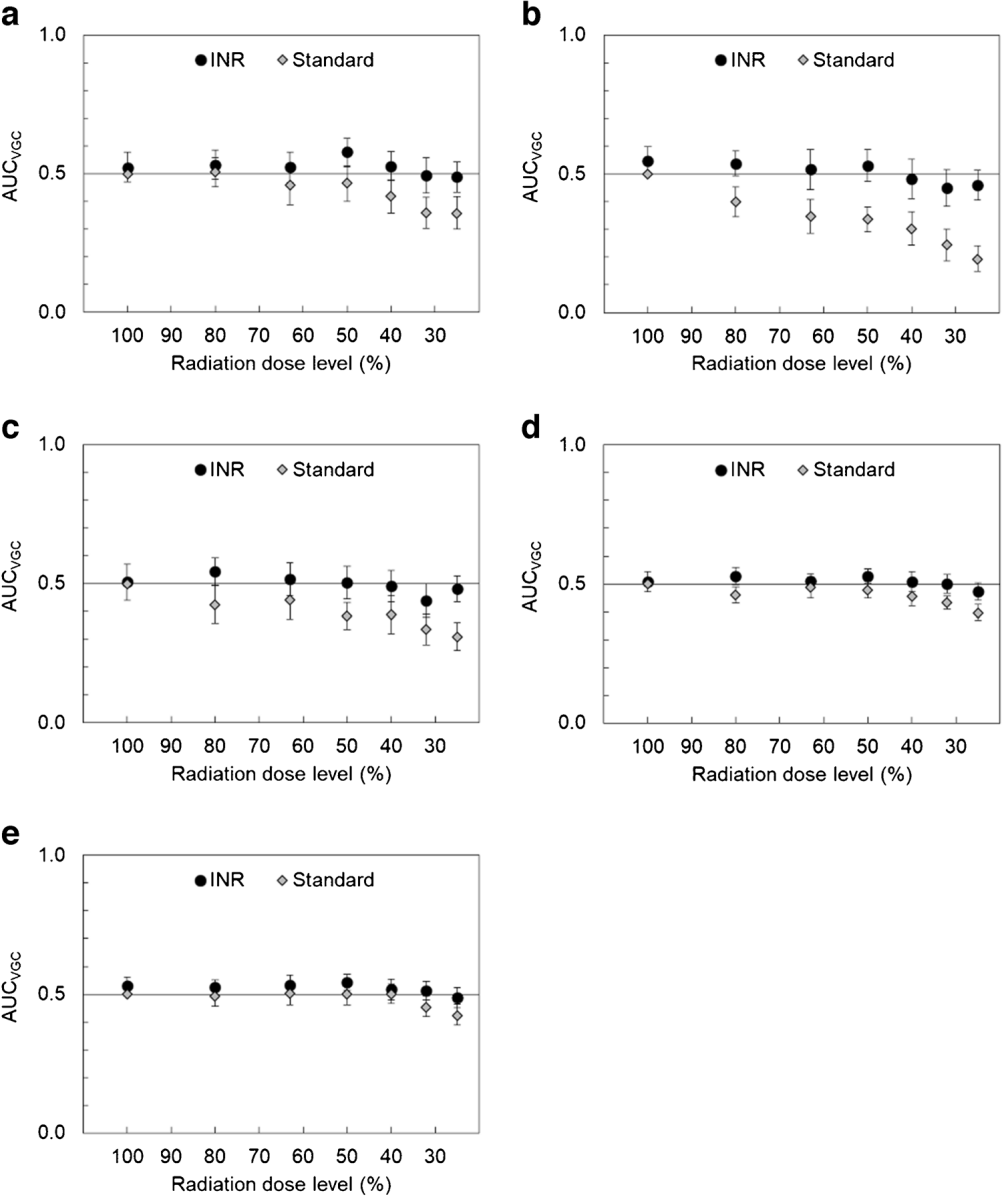
Results of the visual grading characteristic (VGC) analysis for the study comparing lateral chest test images, with intelligent noise reduction (INR) as well as with standard noise reduction, at different dose levels to corresponding reference images (dose level 100% with standard noise reduction). Test images were rated higher than reference images if the area under the VGC curve (AUC_VGC_) > 0.5, and lower if AUC_VGC_ < 0.5. The results were statistically significant if the confidence interval (CI), indicated with *error bars*, did not include 0.5. **a** Visually sharp reproduction of the diaphragm and costo-phrenic angles (criterion L1). **b** Clear reproduction of the spine and paraspinal structures (criterion L2). **c** Clear reproduction of the retrocardiac lung and the mediastinum (criterion L3). **d** Clinically acceptable image quality for a standard lung exam (criterion L4). **e** Clinically acceptable artifacts (criterion L5)

The assessment of the lateral images showed similar trends but with fewer statistically significant differences. The INR images were graded better at dose levels of ≥ 50% compared to the reference images for all criteria L1-L5, though results were not statistically significant; see Fig. 4. The gradings of the INR images at a dose level of 25% were not significantly different from those of the reference images.

Anteroposterior images with standard noise reduction were graded as having statistically significantly lower image quality compared to the reference images at dose levels of ≤ 80% for criteria AP1, AP5, and AP6, ≤ 63% for criterion AP3, and ≤ 50% for criterion AP4 (Fig. 3). There was no statistically significant trend for criterion AP2. The lateral images with standard noise reduction had statistically significantly lower ratings compared to the reference images at dose levels of ≤ 80% for criterion L2, ≤ 50% for criterion L3, and ≤ 40% for criteria L1 and L4 (Fig. 4).

Regarding the grading of artifact level (anteroposterior: criterion AP7, and lateral: criterion L5), no apparent trend was identified for images with INR compared to the reference images at 100% dose level and standard noise reduction (Figs. 3 and 4). Furthermore, the artifact level in the anteroposterior and lateral INR images was graded as significantly better compared to standard noise reduced images at dose levels of ≤ 80% and ≤ 32% (Tables 4 and 5). On the contrary, the artifact level of the standard noise reduced images at the lowest dose levels was graded as significantly impaired compared to the reference image at 100% dose level. A detailed analysis of observer data showed that two observers graded all images with the same artifact level rating (anteroposterior: 1 and 2, lateral: 1 and 3, respectively), while the third observer used a larger span of the rating scale. Free-text comments in ViewDEX showed that the third observer had interpreted artifact level more as noise level, rather than potential artifacts due to INR. No comments regarding actual artifacts in INR images were reported.

## Discussion

In this work, the effect of INR, a newly introduced AI-based software tool for noise reduction in conventional radiography, was studied by evaluating image quality at different dose levels in pediatric chest radiography. Three experienced pediatric radiologists rated the image quality of anteroposterior and lateral pediatric chest images of anthropomorphic phantoms obtained at different dose levels and processed with INR or a standard noise reduction. The image quality of important anatomical structures and the overall diagnostic image quality of the anteroposterior chest images were statistically significantly improved at each investigated dose level, when using INR compared to standard noise reduction. A similar trend of better image quality was seen at all dose levels for the lateral chest images when using INR, although statistically significant improvement was not achieved at the highest dose levels.

INR tended to improve or regain the image quality loss of important structures that followed dose reduction. This was demonstrated in the comparison of the INR images at different dose levels to the reference images at 100% dose level with standard noise reduction. However, the ability of INR to mitigate image quality degradation due to reduced dose varied for different anatomical structures. The visibility of structures and overall diagnostic image quality in anteroposterior INR images were rated significantly better at dose levels of 50–63% and above compared to the reference images, except for the reproduction of spine and paraspinal structures (criterion AP4) where significant improvement was only established at dose levels ≥ 80%. Previous studies on adult chest radiography have shown that the quantum noise in the spinal region has a greater influence on image quality than in other chest regions [25–27]. It is therefore not surprising that it is difficult for INR to regain the image quality in this region as the dose is reduced. Additionally, regarding the clinical impact of this finding, the spine is less important when interpreting pediatric diagnostic chest images compared to the other investigated structures [28]. Therefore, the spine and paraspinal structures may not be a limiting factor in the potential dose reduction of INR, if other, more important, structures such as the vascular pattern, trachea, and the proximal bronchi are reproduced with diagnostic visibility.

INR did not improve the image quality of lateral chest images to the same extent as the anteroposterior chest images. Still, the grading of the lateral images showed trends of enhanced image quality for all structures at dose levels down to 40–50% of the reference images. The image quality at the lowest investigated dose levels – 40% for the anteroposterior and 25% for the lateral INR images – was not statistically significantly different from that of the corresponding reference images. Anteroposterior chest images, which benefitted more from the use of INR, are primarily used for the detection of pathology and are generally of greater clinical importance in pediatrics. Lateral images provide mostly additional information on the position of potential pathology, which may separate malign and benign findings, or tubes and lines in intensive care. Hence, the image quality of lateral images does not have the same requirements regarding details and may not be as sensitive to the radiation dose level as anteroposterior images. The lower degree of improvement with INR for lateral images may be due to a more complex anatomical background and increased anatomical noise arising from the overlay of a larger amount of small lung structures in this projection. This issue might not have been managed to the same extent in the DNN set-up depending on the input images used during the development of INR.

The results of this phantom study indicate that INR improve image quality, also at lower dose levels. The image quality criteria are formulated to reward both low noise and high resolution by the formulation of “clear reproduction” and “visually sharp reproduction” of anatomical structures. INR seemed to reduce noise without degrading other image quality measures, such as spatial resolution, i.e., had the possibility to restore the image quality features of a “high”-dose image in “low”-dose images. Hence, an apparent clinical benefit of applying INR in pediatric chest radiography is the potential to achieve maintained diagnostic image quality at a reduced dose. Generally, the image quality in radiographies performed in radiological department daily should be optimized so that, e.g., the noise level is not interfering with the diagnostic quality, without giving unnecessary high doses to patients. INR seems to have ability to increase the assessed image quality, also in images corresponding to the standard dose level. This may potentially lead to improved diagnostic benefits such as more consistent image quality among the chest radiographies performed and reviewed in a radiological department, by personnel of difference experience. However, it is out of the scope of this study to specify any potential clinical benefits regarding specific pathologies and diagnosis in pediatric chest radiography.

As with other new image processing tools introduced in radiology, limitations, e.g., potential artifacts and loss of diagnostic details, are of interest. In this study, the radiologists graded the diagnostic artifact level in all images. The results from the VGC analysis indicated no significant trends between INR and the standard noise reduced images at dose level 100%; see Figs. 3g and 4e. As described previously, free-text comments in the grading of artifact levels revealed that the slight differences were the result of one radiologist including the noise level in the criterion. This led to impaired grading of the artifact level of the standard noise reduced images at lower doses. No actual artifacts were reported for INR images. Regarding other potential errors, such as loss of anatomical details, this was not specifically evaluated in the present study. However, the radiologists did not comment on any apparent loss of anatomical details in the phantom images, which were quite similar although slightly different projection angles and repeated exposures were used to get different tissue overlap and statistical noise in the images. This question could have been better assessed in a paired study.

Recently, Hussner et al. presented a study evaluating the effect of INR on adult pelvic phantom images and contrast detail radiography phantom images at different dose levels and with different INR levels [29]. The pelvic images were graded by observers in a visual grading analysis. The INR images were reported to have higher image quality than the comparing images with clinical noise reduction. Also, an INR level of 5 to 7 tended to result in the highest score for the pelvic images. The improved image quality in INR images is in accordance with the results of the present study. The INR level in the present study was chosen to have an intermediate effect (5 on a scale from 0–10) on the images, which overlapped with the result of Hussner et al. [29] regarding optimal INR level. However, the optimal INR level may differ between different radiological examinations, patient groups, and clinical questions. Therefore, the optimal choice of INR level for anteroposterior and lateral chest images in pediatric populations needs further investigation.

To the authors’ knowledge, there is only one report on the use of AI-based noise reduction tools in pediatric radiography. Krueger et al. previously applied an AI-based image quality tool for mobile radiography equipment in the pediatric intensive care setting [10]. They observed image quality enhancement of anteroposterior chest and abdominal radiographic images when evaluating the effect of a DNN-based software simulating an anti-scatter grid (SimGrid). Significantly improved image quality was reported when using SimGrid for patients weighing > 10 kg where two experienced pediatric radiologists graded blinded clinical images reconstructed with and without SimGrid. However, the potential for dose reduction was not investigated to avoid a second round of imaging and further exposure of the radiosensitive pediatric patients.

In the current study, the potential dose reduction levels with preserved diagnostic image quality when using INR correspond well with the reported dose reduction interval of 36–70% in the studies reviewed by Ng [6], concerning AI postprocessing tools for dose optimization in pediatric radiology. However, most of these studies investigated the value of DLR for CT images, and only the study by Krueger et al. evaluated pediatric radiographic imaging [10]. The consistent dose reduction results in the present study reinforce the potential impact of AI-based tools for noise reduction and to optimize pediatric radiographic imaging.

The potential radiation dose reduction when using INR is based on the selected reference dose, i.e., the 100% dose level. In this study, the reference dose corresponded to an exposure index resulting in acceptable diagnostic image quality at the present pediatric radiological department. However, the required image quality in pediatric chest radiography may differ between clinical departments and radiologists, e.g., due to experience level. Also, exposure index values vary due to attenuation differences between patients within a certain age- or weight group when using a fixed tube load, often applied for small children. Hence, the study results should be interpreted as indicating the possibility of dose reduction when using INR rather than specifying an absolute magnitude of dose reduction. The grading of the standard noise-reduced images showed trends of decreasing image quality at reduced dose levels corresponding to lower exposure index values. In contrast, INR could produce clinically acceptable images and good reproduction of several important anatomical structures at lower dose levels and exposure index values. This implies that INR may be used as an optimization tool both for radiation dose reduction and to achieve more consistent image quality between different patients, despite the intrinsic variations in exposure index values when using fixed tube load protocols for younger children.

The findings of this phantom study indicate that INR may recover or increase the image quality of pediatric chest radiographs acquired at reduced radiation dose levels. This may decrease patient radiation dose and hence the associated theoretical risk of radiation-induced cancer described by the linear non-threshold model [8]. This model has been strengthened, also for radiation dose levels typically used in diagnostic radiology, by the published results of a large European cohort project [30]. Bosch de Basea et al. reported an increased relative risk of hematological malignancies in children and adolescents due to the radiation dose from CT examinations. Although the radiation dose from a single chest radiography is much lower than that of a CT examination, this reinforces the importance of justifying and optimizing pediatric X-ray examinations. The examinations should be performed according to the as low as reasonably achievable (ALARA) principle, stating that the diagnostic information needed should be obtained with as low a radiation dose as reasonably achievable [7].

The present study was based on the visual grading of anatomical structures in anthropomorphic phantom images. Visual grading of anatomical structures is an established method for analyzing image quality in radiological images [21]. The anatomical structures of interest are typically based on the European guidelines on quality criteria of the diagnostic examination to be considered [19, 31, 32], as in this study. Although no pathology is included, the validity of visual grading studies is described to be high, since the reproduction of clinically important structures in their anatomical background is considered to be related to the probability of discovering pathology in clinical images [21]. Some criteria in the European guidelines include more than one anatomical structure. The combined structures, like spine and paraspinal structures or the retrocardiac lung and the mediastinum, are nearby structures and may be assumed to have the same need of image quality in a chest radiography. In the present study, the radiologists made an overall assessment of the combined structures, like they do in clinical daily basis.

A limiting factor of the present study is that the study was based on anthropomorphic phantom images. However, the anthropomorphic phantoms used in the present study are state-of-the-art phantoms having soft tissue and skeleton substitute materials aiming at patient-like attenuation. Potentially, the phantom images have similar appearance as pediatric patients where no apparent pathology or anatomical anomalies are observed, and may in that aspect, potentially reflect the clinical situation in these cases. However, the anthropomorphic phantoms did not have any pathologies added and the evaluation of image quality was restricted to relevant structures in the chest examination, as discussed previously. Like for all studies based on phantoms, it can therefore be difficult to translate the results to actual patient scenarios and accompanying clinical benefits of specific diagnosis. Furthermore, only two phantoms were used in the study, although this was handled by taking slightly different projections and repetitive exposures of the phantoms. This provided chest images with different anatomical backgrounds, as well as both different anatomical noise and quantum noise, thereby simulating different examinations. Another limiting factor is the relatively small number of radiologists grading the images, leading to the results reflecting their opinions of image quality, which may not be generally applicable to other pediatric radiologists. Despite these limitations, the study was a valuable first step to evaluate the effect of INR in pediatric chest examinations. Further studies are needed to verify the results in clinical pediatric chest radiography.

## Conclusion

The present visual grading study of anthropomorphic phantoms indicates that the AI-based noise reduction algorithm INR may potentially be used to improve image quality or achieve maintained image quality at reduced radiation dose levels in pediatric chest radiography.

## Data Availability

The data sets in the current study are available from the corresponding author on reasonable request.
